# Dietary Fibers (Gum Arabic) Supplementation Modulates Hepatic and Renal Profile Among Rheumatoid Arthritis Patients, Phase II Trial

**DOI:** 10.3389/fnut.2021.552049

**Published:** 2021-03-10

**Authors:** Ebtihal Kamal, Lamis AbdelGadir Kaddam, Alnour Alagib, Amal Saeed

**Affiliations:** ^1^Department of Microbiology and Immunology, Faculty of Medicine, University of Khartoum, Khartoum, Sudan; ^2^Department of Physiology, Faculty of Medicine, Alneelain University, Khartoum, Sudan; ^3^Department of Rheumatology, Military Hospital, Khartoum, Sudan; ^4^Department of Physiology, Faculty of Medicine, University of Khartoum, Khartoum, Sudan

**Keywords:** rheumatoid arthritis, gum acacia, renal function, hepatic profile, dietary fibers, clinical trial

## Abstract

**Background:** Rheumatoid arthritis (RA) is an autoimmune disease that mainly affects the synovial joints with systemic manifestations. RA has a major impact on liver and kidney functions as part of the disease pathogenesis or as a sequel of disease medications or, mostly, both of them. The kidney and liver involvement increases the RA morbidity and mortality. Nowadays, dietary interventions are proposed as potential modifiers for disease severity. Gum Arabic (GA) is acacia senegal exudates; it is soluble fiber with prebiotic properties. GA has been discovered to be protective against experimental nephrotoxicity and hepatotoxicity, with comparable findings in human studies. This article addresses the effect of GA on hepatic and renal profile among RA patients.

**Methods:** Forty patients aged 18–70 received GA daily for 12 weeks as a single dose of 30 g. The liver enzymes, total protein level, serum albumin, serum globulin level, urea, creatinine, and serum electrolytes have been measured as a baseline after 4 weeks and by the end of the study. Cobas C311 (Roche, Germany) automated chemistry analyzer directly determined the values for total protein, albumin, alanine aminotransferase (ALT), aspartate aminotransferase (AST), alkaline phosphatase (ALP), and creatinine. The study ethically has been approved by the Ethical Committee of the National Medicines and Poisons Board. Trial Registration Identifier: NCT02804581.

**Results:** Regarding the liver enzymes, GA has significantly decreased the liver enzymes apart from alkaline phosphatase, which showed no significant change. In contrast, GA has increased the serum albumin level with a minor impact on the serum globulin level. Furthermore, GA has also significantly decreased the level of urea (*P* = 0.0001) and level of Sodium (*P* = 0.002) with nonsignificant change on creatinine and potassium concentrations.

**Conclusion:** GA presents hepatic and renal protective effects among RA patients, evidenced by the significant reduction of urea and liver enzymes. Thus, it can be recommended as a dietary supplement for RA patients. Nonetheless, we recommend further investigation to support our findings.

## Introduction

Rheumatoid arthritis (RA) is an autoimmune rheumatic disorder, a type of systemic disease (1, 2) of unknown etiology (2, 3). Comorbid conditions are common in RA patients either associated with RA itself or related to treatment (RA) (4). The conventional definition of a comorbid condition is a medical condition other than the primary disease itself (5). Comorbidities contribute to a shortened lifespan and life expectancy in comparison to the general population (3). Therefore, comorbidity is a major issue regarding the care of rheumatic disease patients (5).

The most common manifestation of liver damage among RA patients is asymptomatic abnormal liver tests and elevated liver enzymes (4, 6). The main abnormalities are elevated levels of alkaline phosphatase (ALP) and serum gamma-glutamyl transferase (GGT) enzymes (4). The medications used in rheumatology are often hepatotoxic; thus, it is difficult to differentiate between the hepatic manifestations of the primary disease and suspected drug hepatotoxicity (6). Furthermore, patients with autoimmune rheumatic disorders are more susceptible to associated autoimmune liver disease (6), which causes liver damage. The latter may occasionally progress to cirrhosis (6). Rheumatoid arthritis patients have a low level of albumin that is not related to disease activity (7, 8). Hypoalbuminemia may have consequences from a hypercatabolic state secondary to inflammatory status (7), the latter being known as “rheumatoid cachexia” (9). Another supposed mechanism is abnormal albumin distribution between plasma and synovium (8, 10).

Renal disorders are a common cause of mortality among RA patients; however, they are less apparent as clinical manifestations (11). RA patients have a high prevalence of renal impairment with evidence of reduced glomerular filtration and tubular function (12). Nevertheless, renal involvement may remain unnoticed for a long period in a reversible subclinical stage (12). Causes of renal damage among RA patients with advanced stages is difficult to define because of the coexistence of multiple possible causes. Renal Biopsies from RA patients exhibit different histological findings. Most common was mesangial glomerulonephritis (13). Therefore, heterogeneous renal lesions may complicate advanced RA even if they are not clinically obvious (14).

Gum arabic (GA) is a safe dietary fiber approved by the Food and Drug Administration (FDA) and World Health Organization (WHO) (15). It is a high-molecular weight non-starch polysaccharide retrieved from *Acacia senegal* and acacia seyal trees (15, 16). GA is mainly fermented by colonic bacteria instead of being digested by humans (17) and animals (15, 16). Moreover, GA fermentation produces short-chain fatty acids—mainly propionate and butyrate (15–17). The latter was discovered as a physiological modifier for different body functions such as the modulation of cell proliferation, apoptosis, regulation of angiogenesis, and inflammation (18). The anti-inflammatory effect of Gum Arabic has been investigated in various diseases and conditions (19–21). As reported earlier, the effect of GA is an anti-inflammatory agent among RA patients, since it decreases the level of TNF α and the disease severity score among RA patients (22).

Rheumatoid patients are at risk of multi-organ failure, primarily renal insufficiency and hepatic dysfunction even if they are on regular treatment and closely followed up. Anti-inflammatory and analgesic drugs have devastating effects on liver and kidney functions. Therefore, finding safe organ protective agents will be of great benefit for RA patients.

This study aims to investigate the possible ameliorative effect of GA on various parameters of renal and liver function among RA patients.

## Methods and Materials

The study is a single-arm clinical Phase II trial made up of one group of patients. Patients' characteristics and background data have been reported previously (22). Inclusion and exclusion criteria have been described in details earlier (22). In all, 40 participants have completed the study and been included in the final analysis. The main outcome was the level of inflammatory markers and clinical severity score after 12 weeks of GA intake (22). This article documented secondary outcomes considering GA impact on renal and hepatic profile among RA patients.

Thirty grams of GA (acacia senegal exudate), fine powder packed as one sachet, was administered for 12 weeks as a single dose dissolved in water to be consumed in the early morning. Renal and liver function parameters were measured as a baseline, after week 4 and week 12 of GA oral ingestion.

### Measurement of Blood Chemistry

Three ml were withdrawn from a lithium heparin container. Plasma was separated by centrifugation at 2,000 rpm for 10 min and then aliquoted and submitted for biochemical analysis.

Cobas C311 (Roche, Germany) automated chemistry analyzer was used to measure the total protein, albumin, alanine aminotransferase (ALT), aspartate aminotransferase (AST), alkaline phosphatase, amylase, creatinine, total bilirubin, and direct bilirubin values. The machine applies absorption photometry to determine the absorbance amount in the blood that is used to calculate the concentration in the sample and apply the ion-selective electrode (ISE) to measure electrolytes level (23). The concept of measurement of ALT liver enzymes catalyzes the reaction between L-alanine and 2-oxoglutarate (24, 25). While AST catalyzes the transfer of an amino group between L-aspartate and 2-oxoglutarate to form oxaloacetate and L-glutamate (25). The ALP concept of measurementis that, p-nitrophenyl phosphate is cleaved by phosphatases into phosphate and p-nitrophenol in the presence of magnesium and zinc ions. The p-nitrophenol released is directly proportional to the catalytic ALP activity (26).

Data were analyzed using SPSS software package version 23; the quantitative data were expressed as mean, median, standard deviation, minimum, and maximum. Repeated measures ANalysis Of Variance (rANOVA) followed by multiple least significant difference (LSD) comparison tests to detect the changes in biomarker level, *P* ≤ 0.05 were considered significant.

Written informed consent was obtained voluntarily from all participants prior to participation in the study. The study was ethically approved from National Medicines and Poisons Board ERC and registered at ClinicalTrials.gov ID: NCT02804581 Prospective registration.

## Results

**Figure 1:**
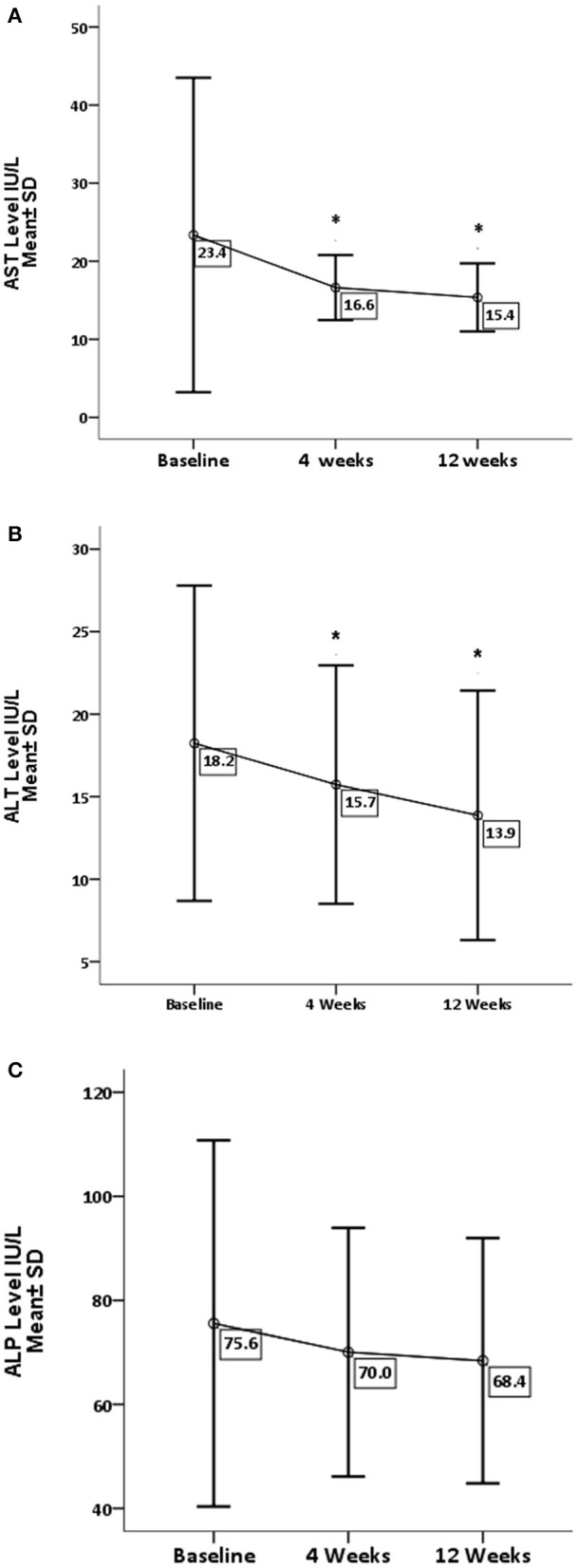
Effect of gum arabic on liver enzymes. *Indicates significant difference from baseline. **(A)** Effect of GA intake on AST Serum Level (*P* = 0.05). **(B)** Effect of GA intake on ALT Serum Level (*P* = 0.001). **(C)** Effect of GA intake on ALP Serum Level (*P* = 0.209).

**Figure 2:**
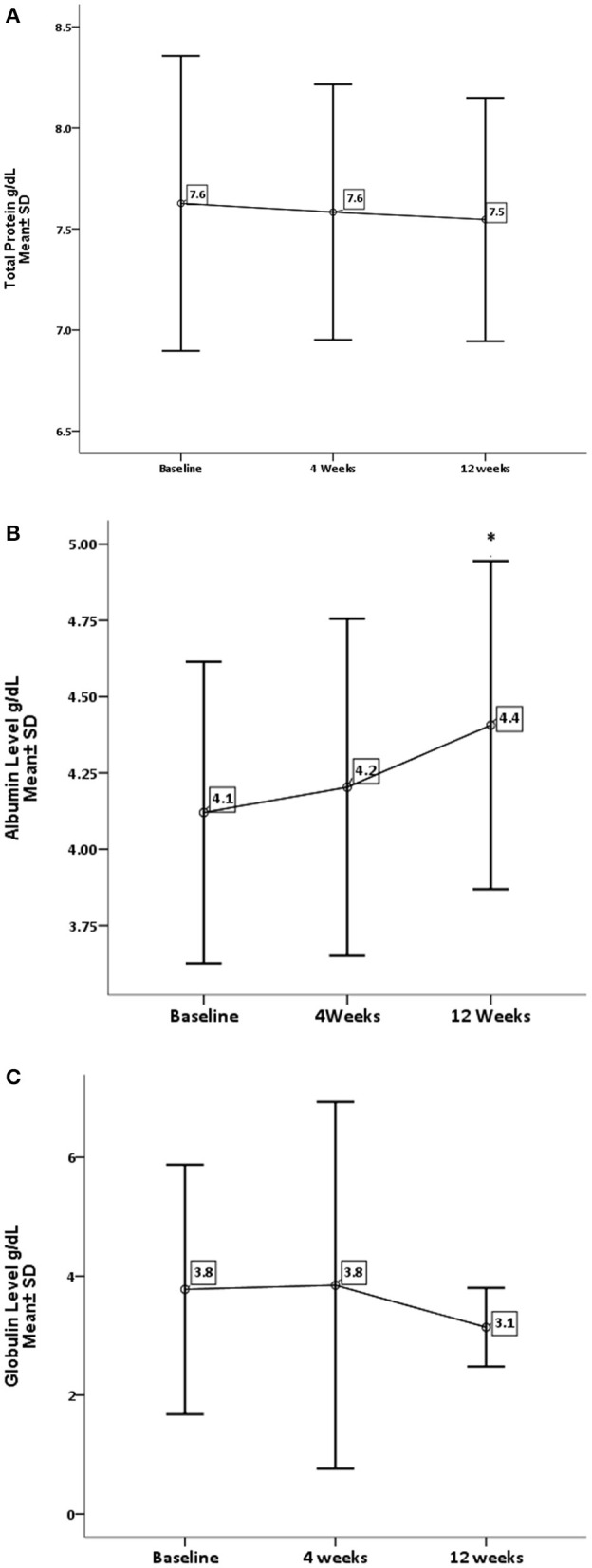
Effect of gum arabic on liver function test. *Indicates significant difference from baseline. **(A)** Effect of GA intake on total proteins level (*P* = 0.543). **(B)** Effect of GA intake on albumin level (*P* = 0.019). **(C)** Effect of GA intake on globulin level (*P* = 0.222).

Table 1 represents baseline values of renal and hepatic profiles. As for the effect of GA on liver enzymes (Figure 1), there is a significant decrease in AST and ALT levels, with *P*-values 0.05 and 0.001, respectively. While there is no significant difference in ALP (*P* = 0.209), GA has significantly increased the albumin level (*P* = 0.019) with no effect on the total bilirubin and globulin levels (Figure 2).

**Table 1:** Age and baseline characteristics of the biochemical profile.

**Characteristics**	**Mean**	**Median**	**SD**	**Minimum**	**Maximum**
Age (years)	48.25	51.00	12.54	18	70
AST enzyme level (U/l)	23.03	18.0	19.89	10.0	119.0
ALT enzyme level (U/l)	18.16	15.0	9.399	6.0	50.0
ALP enzyme level (U/l)	76.59	70.00	32.14	40	216
Total Protein level (g/dl)	7.6	7.8	0.72	6.0	8.9
Albumin level (g/dL)	4.13	4.0*	0.49	2.90	5.0
Serum Globulin level (g/dl)	3.75	3.30	2.07	1.10	14.0
Urea level (mg/dl)	25.87	23.00	11.14	14.0	62.0
Creatinine level (mg/dl)	0.74	0.70	0.31	0.30	2.0
Sodium Level (mmol/l)	140.81	141.0	3.69	131.0	148.0
Potassium (mmol/l)	3.93	3.8	0.96	3.30	8.90

**Figure 3:**
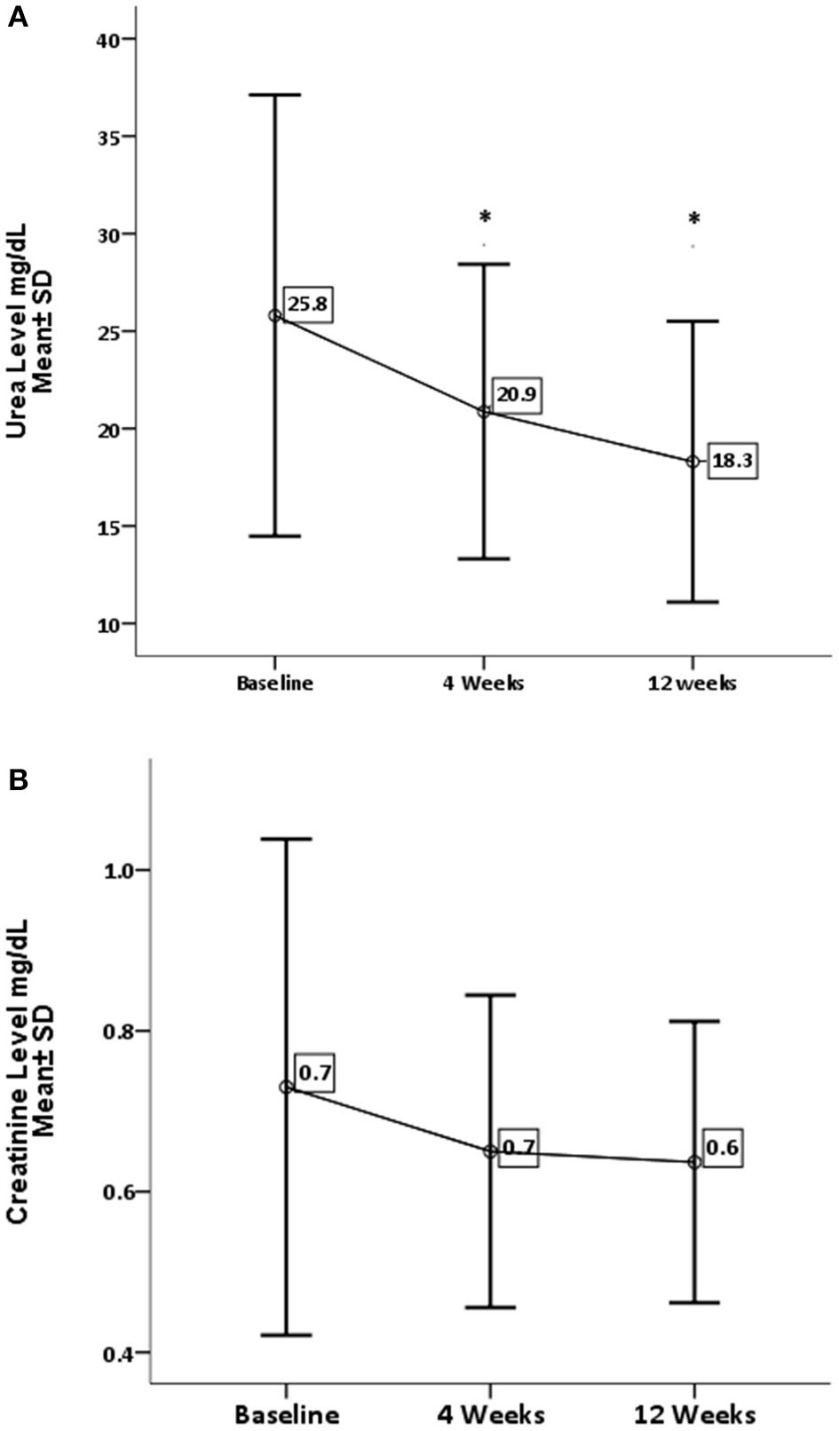
Effect of gum arabic on renal profile. *Indicates significant difference from baseline. **(A)** Effect of GA intake on urea serum level (*P* = 0.0001). **(B)** Effect of GA intake on creatinine level (*P* = 0.111).

**Figure 4:**
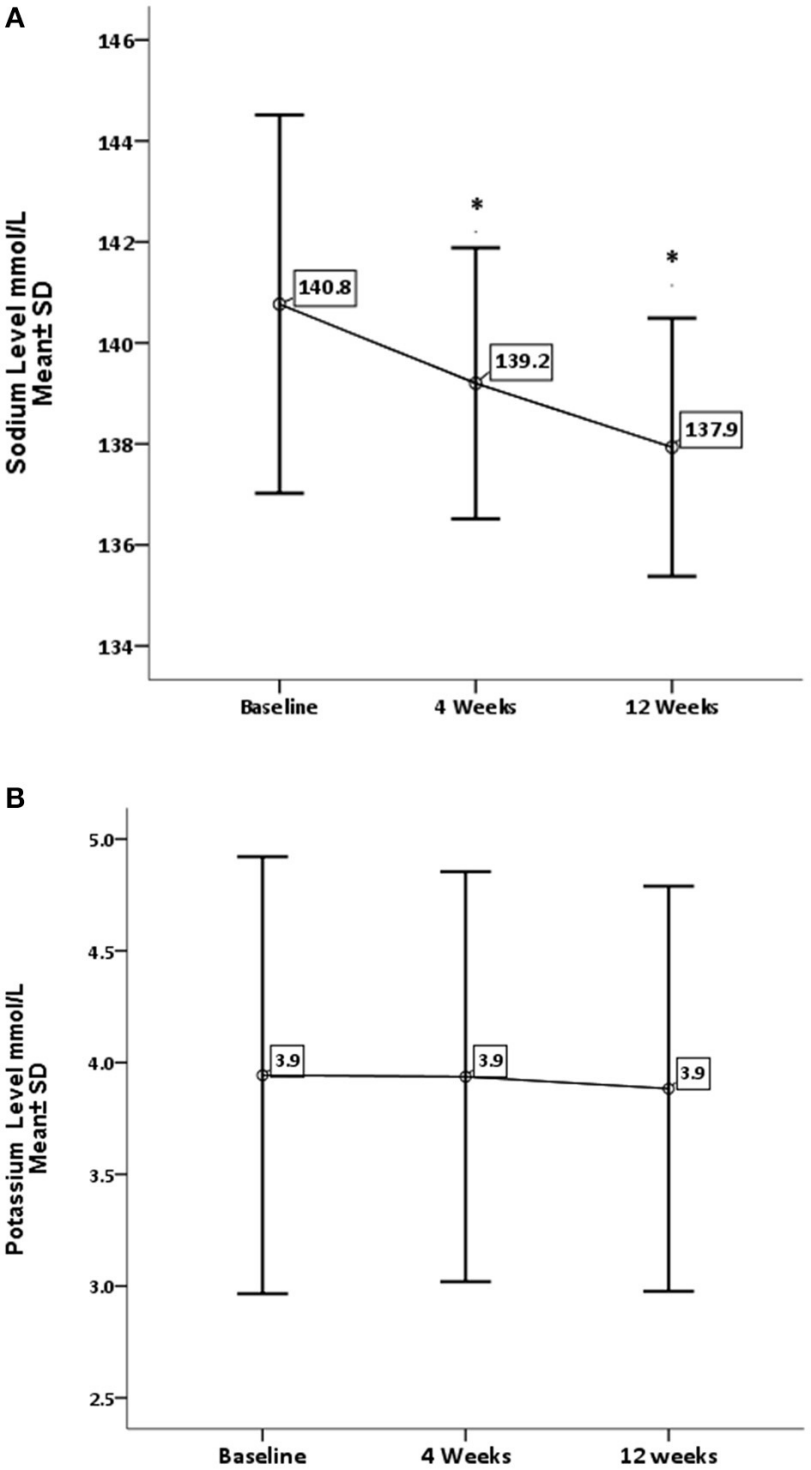
Effect of gum arabic on serum electrolytes. *Indicates significant difference from baseline. **(A)** Effect of GA intake on sodium level (*P* = 0.002). **(B)** Effect of GA intake on sodium level (*P* = 0.383).

Concerning renal function and electrolytes level, GA has significantly decreased the level of urea (*P* = 0.0001) and sodium (*P* = 0.002). On the other hand, GA shows no influence on creatinine and potassium levels, as shown in Figures 3, 4.

## Discussion

Evaluation of renal and hepatic function is necessary since many antirheumatic agents cause renal or hepatic toxicity and may be contraindicated if these organs are impaired (1). So these parameters were measured regularly during the clinical trial.

The current study reveals that GA can significantly decrease liver enzymes apart from ALP (Figure 1). These findings are consistent with previous animals and human studies, and earlier animal experiments had documented the favorable effect of GA on liver enzymes (27, 28). Furthermore, GA has decreased ALT level among healthy subjects and sickle patients (29, 30). Nowadays, there is strong evidence confirming the significant role of the liver in the modulation of immune response in autoimmune and chronic inflammatory disorders (6). GA showed no effect on ALP, which is consistent with earlier research (30). Elevated ALP is hepatic in origin among at least one-third of patients (6), while ALT is a well-recognized biomarker for drug-induced hepatotoxicity (31). These findings confirm the hepatoprotective effect of GA consumption, which increases albumin levels with no significant increase in globulin or total protein as seen in Figure 2. These results contradict a previous study when GA did not alter the albumin level among sickle cell patients (30). The elevation of albumin levels could be secondary to the anti-inflammatory effect of GA (22), which may decrease the catabolic state among RA patients. The latter was proposed as a cause of hypoalbuminemia among RA patients (7). With respect to other liver function parameters, GA did not alter total protein and globulin levels, which is consistent with the previous study (30).

RA has a destructive effect on kidney functions, chronic kidney disease is a common presentation among RA patients (32, 33). The inflammation proposed as a risk factor for CKD among rheumatoid patients (32, 33). The beneficial effect of GA on renal functions is one of the early and documented studies among both humans and rats (34). GA in the current study has significantly reduced urea levels with no impact on creatinine levels (see Figure 3). These findings are comparable to the GA effect among sickle cell patients (30). GA increased nitrogen excretion in stool reducing serum urea levels (34, 35); probably this is what reduced the urea levels in our study, in addition to the reduction in inflammatory status due to regular GA consumption (22). The creatinine clearance was found to be low among RA patients compared with healthy subjects because of muscle atrophy and lower creatinine production (11). Moreover, most of our patients' creatinine levels were within normal limits, as shown in Table 1, which might explain why GA did not have an effect on creatinine in the current research (Figure 3). GA improved renal profiles among chronic renal failure patients and corrected renal dysfunction secondary to induced nephrotoxicity (36–38). Besides, GA acts as an anti-inflammatory and antioxidant among end-stage renal patients (21). As for serum electrolytes, GA significantly reduced sodium levels with no significant effect on potassium (see Figure 4). The reduction in sodium may protect against the development of hypertension, since RA patients are at risk of developing hypertension, which is underdiagnosed (39) and associated with asymptomatic organ damage (40). An experimental study revealed that GA intake increased sodium excretion in urine without affecting serum level (41). Previous clinical trials showed GA did not affect electrolyte levels (29, 30). An earlier study showed that the rheumatoid patients had lower salivary potassium levels compared with healthy subjects (42).

RA is an inflammatory disease affecting mainly synovial joints; nevertheless, the disease pathogenesis and treatment side effects take a tremendous toll on liver and kidney functions. GA, which is a soluble dietary fiber, exhibits potent anti-inflammatory activity among rheumatoid patients; also, it displays strong renal and hepatic protective effect evidenced by a significant decrease in liver enzymes and urea levels. The limitations of our study are that it was a single-arm and short-time study (only 12 weeks). Since it was conducted, it is the first study ever to investigate GA organ protective properties among RA patients.

## Conclusion

In conclusion, our results document the potential efficacy and safety of Gum Arabic (*A. senegal*) as a nutritional supplementary agent for the management of RA supported by the reduction of ALT, AST, and urea serum level and improved level of albumin. Nevertheless, our findings should be validated further by a long-term randomized placebo-controlled trial to establish its utilization in the clinical setting.

## Data Availability Statement

The original contributions presented in the study are included in the article/Supplementary Material, further inquiries can be directed to the corresponding author/s.

## Consent for Publication

The principal investigator obtained informed consent from each participant to publish the data without breaching confidentiality.

## Ethics Statement

The studies involving human participants were reviewed and approved by National Medicines and Poisons Board Ethics Research Committee. The patients/participants provided their written informed consent to participate in this study.

## Author Contributions

EK, LK, and AS participated in study design. EK and LK, were involved in all aspects of the study conduct. EK and LK participated in the data collection process. EK and LK analyzed the data. LK and EK participated in the writing and reviewing the manuscript. AA was significant clinical contributor to the study. All authors approved final version of manuscript.

## Conflict of Interest

The authors declare that the research was conducted in the absence of any commercial or financial relationships that could be construed as a potential conflict of interest.

## Abbreviations

ALP: Alkaline Phosphatase
ALT: Alanine Transaminase
AST: Aspartate Transaminase
GA: Gum Arabic
LFT: Liver Function Test
RA: Rheumatoid Arthritis
RFT: Renal Function Test.
