# Supplementary figures and images for: Dietary Fibers (Gum Arabic) Supplementation Modulates Hepatic and Renal Profile Among Rheumatoid Arthritis Patients, Phase II Trial
Source: Front Nutr. 2021 Mar 10;8:552049. doi: 10.3389/fnut.2021.552049 (PMC7987669; doi:10.3389/fnut.2021.552049)

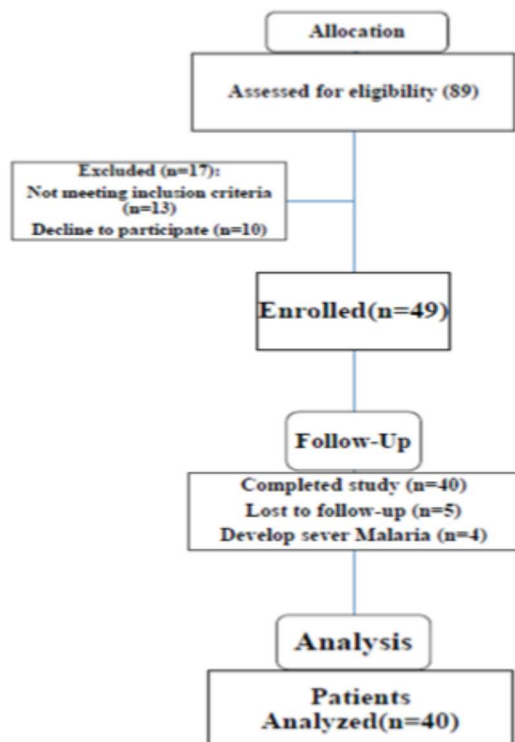

Supplement: Supplementary file 1 [file Data_Sheet_1.PDF]
